# Immune Cells from SR/CR Mice Induce the Regression of Established Tumors in BALB/c and C57BL/6 Mice

**DOI:** 10.1371/journal.pone.0059995

**Published:** 2013-03-21

**Authors:** Janne Koch, Jann Hau, Jan Pravsgaard Christensen, Henrik Elvang Jensen, Morten Bagge Hansen, Klaus Rieneck

**Affiliations:** 1 Department of Experimental Medicine, Faculty of Health Sciences, University of Copenhagen, Copenhagen, Denmark; 2 Department of International Health, Immunology and Microbiology, Faculty of Health Sciences, University of Copenhagen, Copenhagen, Denmark; 3 Department of Veterinary Disease Biology, Faculty of Life Sciences, University of Copenhagen, Frederiksberg, Denmark; 4 Department of Clinical Immunology, Rigshospitalet, University Hospital of Copenhagen, Copenhagen, Denmark; University of Nebraska Medical Center, United States of America

## Abstract

Few experimental models are available for the study of natural resistance to cancer. One of them is the SR/CR (spontaneous regression/complete resistance) mouse model in which natural resistance to a variety of cancer types appeared to be inherited in SR/CR strains of BALB/c and C57BL/6 mice. The genetic, cellular, and molecular effector mechanisms in this model are largely unknown, but cells from the innate immune system may play a significant role. In contrast to previous observations, the cancer resistance was limited to S180 sarcoma cancer cells. We were unable to confirm previous observations of resistance to EL-4 lymphoma cells and J774A.1 monocyte-macrophage cancer cells. The cancer resistance against S180 sarcoma cells could be transferred to susceptible non-resistant BALB/c mice as well as C57BL/6 mice after depletion of both CD4+/CD8+ leukocytes and B-cells from SR/CR mice. In the responding recipient mice, the cancer disappeared gradually following infiltration of a large number of polymorphonuclear granulocytes and remarkably few lymphocytes in the remaining tumor tissues. This study confirmed that the *in vivo* growth and spread of cancer cells depend on a complex interplay between the cancer cells and the host organism. Here, hereditary components of the immune system, most likely the innate part, played a crucial role in this interplay and lead to resistance to a single experimental cancer type. The fact that leukocytes depleted of both CD4+/CD8+ and B cells from the cancer resistant donor mice could be transferred to inhibit S180 cancer cell growth in susceptible recipient mice support the vision of an efficient and adverse event free immunotherapy in future selected cancer types.

## Introduction

Mouse strains that survive injection of large numbers of cancer cells are rare [1]. Such mice constitute important experimental models for cancer resistance at the cellular and molecular levels. The spontaneous regression/complete resistance (SR/CR) mice were derived from BALB/c mice and described by Cui and colleagues in 1999 [2]. The phenotype was characterized by the ability to resist challenges from a number of cancer cell lines [3], [4]. This resistance involved innate immune cells, including polymorphonuclear granulocytes (PMNs), macrophages, and NK cells [4], [5]. The SR/CR phenotype was inherited by approximately 30% of the offspring when SR/CR mice were mated with wild-type mice of the parental strain [6].

Interestingly, adoptive transfer (AT) of SR/CR leukocytes rendered recipients resistant to the intraperitoneal (i.p.) injection of S180 cells and also induced the regression of solid tumors. Yet, no systematic immunohistochemical analysis of the hypothetical interplay between cancer cells and the putative immune effector cells in the tumor tissue has been performed. Hematoxylin and eosin (HE) staining suggested that the tumor tissue might be surrounded by PMNs and macrophages, and the periphery might contain increased numbers of plasma cells [5].

In this study we tested whether the cancer resistance of the SR/CR mice could be transferred to cancer susceptible mice by AT of selected immune cells. All experiments were adequately powered and clinical outcomes were supplemented with appropriate immunohistochemical analyses.

## Materials and Methods

### Mice

BALB/c and C57BL/6 mice (Charles River, Sulzfeld, Germany) were either purchased or bred from breeding pairs at the Department of Experimental Medicine, University of Copenhagen.

SR/CR mice on BALB/c and C57BL/6 backgrounds were a generous gift from Dr. Zheng Cui, Wake Forest University, North Carolina. These mice were bred at the Department of Experimental Medicine, and the progeny that tested positive for the SR/CR phenotype were used in the described experiments. Genders of the control BALB/c and C57BL/6 mice were matched with the genders of the SR/CR mice.

All mice were group-housed in IVC racks (Tecniplast, Varese, Italy) in ventilated polycarbonate type III cages (Tecniplast) under standard conditions: 12 hour artificial light-dark cycles, a temperature of 21±1°C and a relative humidity of 30–60%. The bedding material was composed of Aspen chips (Tapvei Oy, Kortteinen, Finland) and shredded cardboard, and cardboard hides were used for environmental enrichment. Acidified tap water and a standard rodent pellet diet (Altromin 1319, Brogaarden, Gentofte, Denmark) were provided *ad libitum*. All laboratory animal work was conducted in accordance with Danish legislation and was approved by the Animal Experiments Inspectorate (under the Danish Ministry of Justice), and all animals were inspected daily by trained animal technicians. The experiments were performed under the following license: 2005/561-1059.

The experimental animal work was performed at the Department of Experimental Medicine of the University of Copenhagen, Denmark, which is accredited by the Association of Assessment and Accreditation of Laboratory Animal Care (AAALAC).

### Tumor Cell Lines and Culture

The S180 sarcoma cell line (ATCC, Boras, Sweden) was tested negative for all relevant murine viruses and mycoplasma using a MAP full-panel test (Taconic, Germantown, NY, USA) prior to use. EL-4, J774A.1 (ATCC) and S180 cell lines were maintained in cell culture until *in vivo* transplantation. The cells were cultured in DMEM with GlutaMAX-I and HEPES (Invitrogen, Taastrup, Denmark), as previously described (7). Penicillin and streptomycin (Invitrogen) were added to a final concentration of 100 IE/ml and 100 µg/ml, respectively, and fetal calf serum (FCS) (Invitrogen) was added to a final concentration of 10%. The medium was changed every second day, and the cells were seeded at a density of 4-5×10^5^ viable cells/ml. The cells were split when reaching a density of 1-2×10^6^ viable cells/ml. Cells were frozen in 10% DMSO (Sigma-Aldrich, Brøndby, Denmark), 20% FCS and 70% DMEM (Invitrogen) at a density of approximately 5×10^6^ cells/ml and were stored in liquid nitrogen. During the screening procedures, S180 cells were maintained in the ascitic fluid of BALB/c or C57BL/6 mice (Charles River, Germany).

The YTA, YTS 191, YTS 156 and YTS 169 hybridoma cell lines [7], [8] were cultured in Protein-free Hybridoma Medium (PFHM-II) (Invitrogen) with 0.1% penicillin/streptomycin (Invitrogen). The antibodies were harvested from the media when the cells reached appropriate densities. YTA and YTS 191 produced anti-CD4 rat antibody, and YTS 156 and YTS 169 produced anti-CD8 rat antibody.

### *In Vivo* Cell Maintenance

Initially, ascites was induced in BALB/c and C57BL/6 mice by the i.p. injection of 2×10^5^ S180 cells from an *in vitro* culture. After approximately two weeks, the mice developed ascites and were euthanized by cervical dislocation. The ascitic fluid was immediately removed by aspiration with a 10 ml syringe and a 19 G needle. The cells were then washed twice in 10 ml of sterile PBS and were counted in a hemocytometer (Reichert, Depew, NY, USA) using Trypan Blue (Sigma-Aldrich) in a 1:1 ratio with the cell suspension. The cells were adjusted to 200,000 cells/ml in sterile PBS, kept at room temperature and used for i.p. injection within one hour. The tube containing the cells was inverted several times to maintain the cells in suspension. The mice were injected i.p. with 1 ml of the cell suspension using a 25 G needle on a 1 ml syringe and were returned to their cage immediately after injection. This procedure was repeated to maintain the S180 cells under *in vivo* culture conditions. Throughout the experiments, gender and strain barriers were never crossed.

The J774A.1 cells were injected i.p. in a 1 ml suspension with sterile PBS at a concentration of 8×10^5^ cells/ml. Because J774A.1 cells have been documented to induce ascites in all of the C57BL/6 mice tested, only SR/CR mice in a C57BL/6 background were tested for resistance to this cancer cell line(10).

### Induction of Subcutaneous Tumors by the Transplantation of Solid EL-4 Tumor Pieces or by the Injection of Tumor Cells

Mice were anesthetized with a subcutaneous (s.c.) injection of a mixture of Hypnorm (10 mg/ml) (VetPharma Ltd, Leeds, UK), midazolam (5 mg/ml) (Hameln Pharmaceuticals, Hameln, Germany) and sterile water (1∶1∶2). The tumor transplants had a size of approximately 1 mm^3^.

A 1∶1 analgesic mixture of bupivacaine (10 mg/ml) and lidocaine (10 mg/ml) (SAD, Region Hovedstaden Denmark) was injected s.c. prior to the skin incision.

A 5 mm skin incision was created over the vertebra in the region between the scapulae. Using blunt dissection, subcutaneous pockets were created in each axillary region, and the tumor pieces were placed into the distal region of these pockets. The skin incision was then closed with Nexaband tissue adhesive (Jørgen Kruuse A/S, Langeskov, Denmark). When inducing tumors by injection of tumor cells a 0.1 ml suspension of 10^6^ EL-4 or S180 cancer cells in sterile PBS was injected s.c. into each axilla. The tumors were injected into eight SR/CR mice on C57BL/6 background and into eight C57BL/6 mice. Half of the mice from each group were inoculated with transplants and the other half with EL-4 cell suspensions.

### Adoptive Transfer (AT) of Leukocytes and Depletion of CD4+/CD8+ T Cells and B Cells from Splenocytes and Peritoneal Exudate Cells to Recipients with S180 Tumors

Two days prior to the isolation of immune cells, SR/CR mice and control C57BL/6 donor mice were injected i.p. with 2×10^6^ irradiated S180 cells. Splenocytes and peritoneal exudate cell mixtures were produced according to the following procedure: Immune cells were harvested from aseptically removed spleens by pressing the cells through a sterile mesh. The cells were washed twice in 10 ml of HBSS (The Panum Institute, Copenhagen) equilibrated to room temperature, centrifuged at 1250 rpm for 5 minutes and counted. Peritoneal immune cells were isolated by flushing the peritoneal cavity with 5 ml of sterile PBS and retrieving 80–90% of the volume. This suspension was washed and centrifuged twice at 1250 rpm for 5 minutes in sterile PBS, and the cells were counted before being mixed with the splenocyte suspension from the same donor mouse. The resulting mixture was resuspended in 2 ml of sterile PBS prior to i.p. injection. When S180 tumors had reached a size of approximately 100 mm^3^, mice were injected i.p. with leukocytes from the spleens and peritoneal fluid of SR/CR mice or nonresistant controls at a 1∶1 ratio (i.e., one donor mouse per recipient mouse). The leukocytes were transferred from eight SR/CR mice on C57BL/6 background and from seven C57BL/6 mice in the control group. Strain and gender barriers were never crossed during the transfers.

Tumor development was inspected daily, and tumors were measured every second day using calipers. The tumor volume was calculated using the formula (b x d x h)/2, where b, d and h were the base, diameter and height. When the longest diameter of the tumors reached 12 mm, the mice were euthanized according to the conditions of the license (see below).

In cases of regressing tumors, one tumor was removed unilaterally during anesthesia, as described in the transplantation section above, to confirm that the remaining tumor regressed. Mice with complete tumor regression were injected i.p. with 2×10^6^ S180 cells 3 weeks later and were carefully observed for 60 days.

Rat antibodies produced by YTA and YTS191 cells were used to deplete CD4+ cells, and rat antibodies produced by YTS 156 and YTS 169 cells were used to deplete CD8+ cells, as previously described [7], [8]. Briefly, after the spleen and peritoneal cell suspensions were prepared, hybridoma cell-derived antibodies were added, and the cell suspensions were incubated 30 min on ice and were washed twice in 10 ml of sterile PBS to remove unbound antibodies. After the cells were suspended in Hanks BSS, Dynabeads (Invitrogen) against rat IgG were added to remove the YTA-, YTS 191-, YTS 156- and YTS 169-coated cells. Dynabeads against mouse IgG were added to remove the B cells via membrane binding to IgG antibodies. This suspension was incubated for 40 min on a blood rotator. The beads were removed using a magnetic stand, the suspended cells were washed twice in 5 ml of HBSS at room temperature and suspended in 5 ml of sterile PBS, and aliquots from the suspension were prepared for FACS analysis. Leukocytes were counted before and after depletion, and in all instances, between 1×10^7^ and 2×10^8^ leukocytes were transferred to the S180 cell tumor-bearing recipients in a 1∶1 ratio. The depleted leukocytes were removed from either six SR/CR mice (three on C57BL/6 background and three on BALB/c background) or six donor mice (three C57BL/6 and three BALB/c). Strain and gender barriers were never crossed during the transfers. Mice exhibiting complete tumor regression were injected i.p. with 2×10^6^ S180 cells three weeks after the transfers and were carefully observed for 60 days.

### FACS Analyses

The leukocytes were suspended in FACS media consisting of PBS containing 10% rat serum and 10% BSA (Sigma-Aldrich) and were analyzed on a BD FACSCanto II flow cytometer (BD Bioscience, Mountain View, CA, USA). The cells were prepared according to the manufacturer’s recommended protocol. FACS was used to assess the depletion of CD4+/CD8+ T cells and B cells and to quantify the fractions of the innate immune cell subpopulations transferred to each tumor-bearing mouse. The following antibodies were used: anti-CD45, -CD16/32, -CD3, -CD4, -CD19, -F4/80 and NK1.1 (BD Pharmingen, San Diego, CA, USA), isotype controls and an anti-PMN antibody (ab53453, Abcam, Cambridge, UK). Cells from the non-depleted suspensions were prepared in parallel to assess the level of depletion.

### Myeloperoxidase, CD3 and Caspase-3 Staining of Tumor Tissue Sections

Following euthanasia, tumor tissue was removed aseptically and immediately fixed in a 4% formalin buffer (Bie & Berntsen A/S, Rødovre, Denmark) at room temperature for two days. The fixed tumors were processed through graded concentrations of ethanol and xylene and were then embedded in paraffin wax. Tissue sections of 4–5 µm were mounted on adhesive glass slides (Thermo Scientific, Menzel GmbH & CoKG, Baunschweig, Germany) and were stained with HE.

For the myeloperoxidase staining, the sections were deparaffinized through graded alcohol solutions. Antigen retrieval was performed using Diva Decloaker buffer (Biocare Medical, LLC, CA, USA) in a 2100 Retriever (PickCell Laboratories, Amsterdam, the Netherlands) according to the manufacturer’s protocol. The tissue slides were blocked in normal goat serum (NGS), avidin (Bie & Berntsen) and biotin (Bie & Berntsen); subsequently, they were incubated overnight with an anti-human myeloperoxidase antibody (A0398, Dako, Glostrup, Denmark) at a 1∶5000 dilution in Tris-buffered saline, pH 7.6 (TBS). The slides were then incubated for 1 hour with the secondary antibody from the Elite ABC kit (Bie & Berntsen), the label complex from the kit was added, and the staining was visualized by a reaction with DAB for 6 min in the dark. CD3 staining was performed using the previously described procedure, and the primary CD3 antibody (Clone SP7; Thermo Scientific, Copenhagen, Denmark) was added at a 1∶1600 dilution in TBS. Subsequently, the same reagents were added as described above for the myeloperoxidase staining.

Prior to staining with the caspase-3 antibody, sections were deparaffinized and blocked with endogenous peroxidase in 0.6 ml H_2_O_2_ for 15 minutes, followed by blocking with Ultra V Block for 5 min. The primary anti-caspase-3 antibody (#9661; Cell Signaling Technology, Inc., MA, USA) was diluted 1:750 with TBS and was incubated overnight. The UltraVision ONE HRP polymer (LabVision Corporation, CA, USA) was added for 30 min, and the staining was visualized using an amino-ethyl-carbazole (AEC-red) single solution for 10 min, as described by the manufacturer (LabVision Corporation). After the immunostaining, the sections were counterstained with Mayer’s hematoxylin (Sigma- Aldrich), flushed in distilled water and mounted. Throughout the immunostaining protocols, with the exception of the step between the blocking of unspecific binding with biotin or Ultra V Block and the application of the primary antibodies, the slides were washed in TBS.

### Statistical Analyses

The differences between the numbers of leukocytes from the SR/CR mice and the nonresistant/susceptible control mice were judged using a two-sided, unpaired Student’s t-test with a significance level of 5%. The differences in tumor size between the mice receiving AT from SR/CR mice and susceptible controls were judged using one-way ANOVA tests with a significance level of 5 %. The tests were performed with GraphPad Prism, version 5 (GraphPad Prism Software, La Jolla, CA, USA).

## Results

We investigated the responses and the histological features of cancer resistant SR/CR mice and cancer susceptible control mice to different cancer cell lines. In addition, we examined to what extent AT of total leukocytes and leukocytes depleted of adaptive immune cells derived from SR/CR mice could induce the regression of tumors in susceptible recipient mice.

### Tumor Growth in C57BL/6 Mice with or without SR/CR Phenotypes

**Figure 1:**
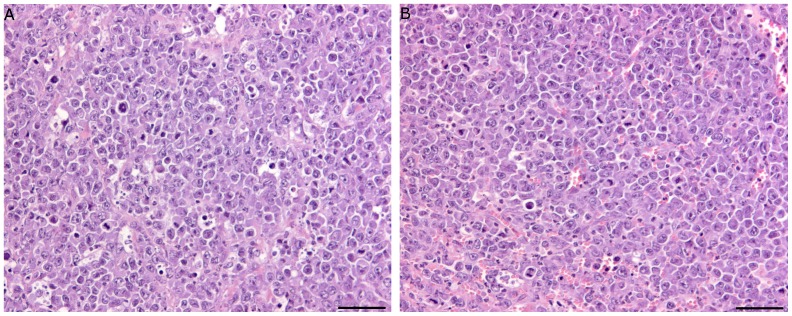
EL-4 tumors grew in both SR/CR cancer resistant mice and C57BL/6 cancer susceptible mice. No immune cell infiltration was observed in EL-4 tumor tissues from SR/CR cancer resistant mice (A) or C57BL/6 cancer susceptible mice (B) transplanted with EL-4 tumor pieces two weeks earlier (representative sections, n = 8). EL-4 tumor tissue contained mitotic figures and virtually no infiltration of immune cells (HE stain, bar 100 µm).

**Figure 2:**
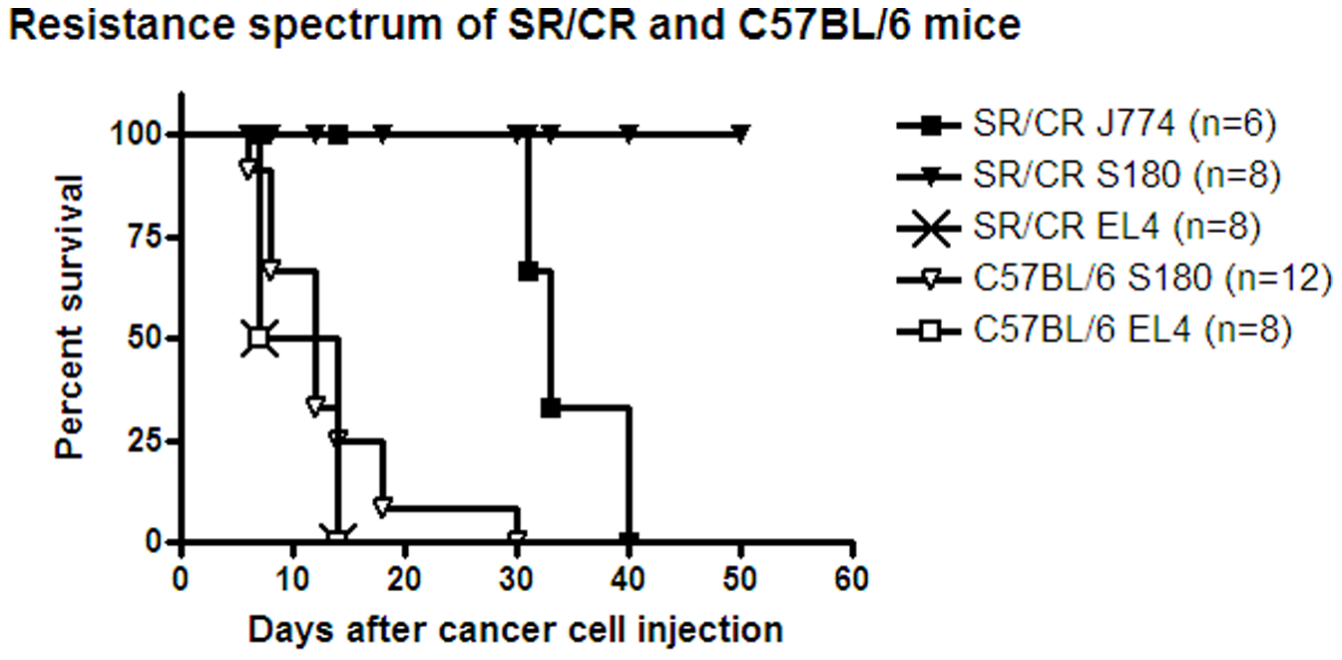
Cancer growth susceptibility and resistance of SR/CR mice and nonresistant controls. The SR/CR mice resisted the s.c. injection of S180 cells, whereas the C57BL/6 mice developed tumors 8–30 days after s.c. injection of S180 cells which was at a significantly higher frequency (p<0.0001). SR/CR and C57BL/6 mice both developed tumors 8–14 days after the transplantation or s.c. injection of EL4 cells. SR/CR mice were inoculated i.p. with 8×10^5^ J774A.1 cells and ascites developed 34–40 days later, and this resistance was significantly lower (p<0.0081) compared to the resistance of SR/CR mice to S180 cells.

All of the SR/CR mice on C57BL/6 background (n =  8) and all of the susceptible C57BL/6 control mice (n =  8) developed EL-4 cell tumors within one week after transplantation. Within 1–2 weeks of transplantation or s.c. injection into SR/CR mice, the tumors grew to sizes that required euthanasia. Tissue sections from all mice showed no leukocyte infiltration in the tumor tissues (Figure 1). In addition, six out of eight SR/CR mice developed cancer and severe ascites due to cancer cell growth 30–40 days after the i.p. injections of J774A.1 cells (Figure 2).

**Figure 3:**
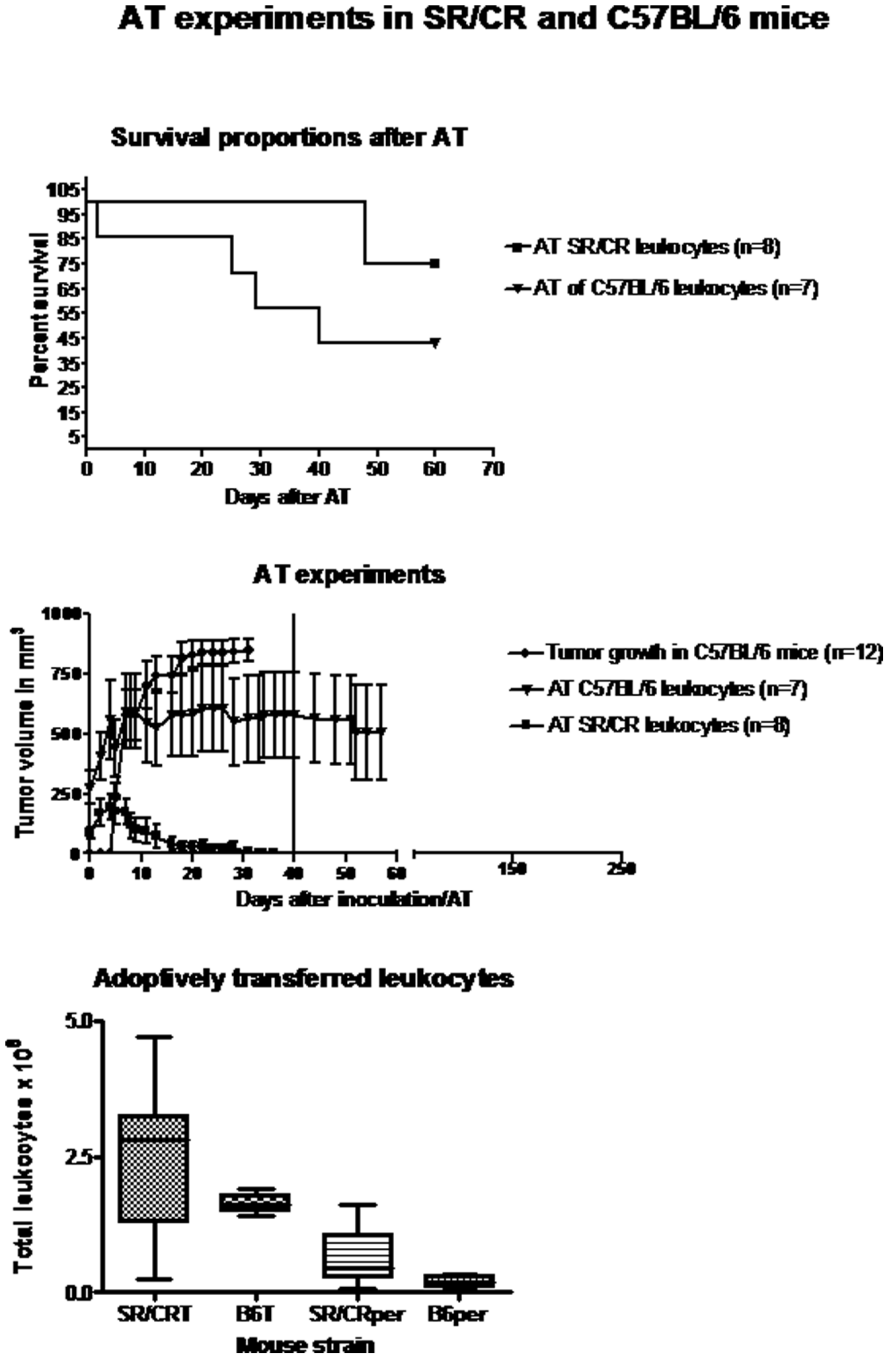
Differences in tumor regression in S180-cancer susceptible C57BL/6 mice after the AT of leukocytes from either cancer resistant SR/CR mice or non-resistant controls. (A) Survival of S180-cancer susceptible C57BL/6 mice after AT. Two mice that received SR/CR-leukocytes and four mice that received C57BL/6 leukocytes were euthanized due to excessive S180 tumor burdens. (B) Tumor volumes after AT. Progressive tumor growth was observed in untreated C57BL/6 mice after the bilateral s.c. injection of 10^6^ S180 cells in the axillary region. The growth reached a plateau approximately 20 days post tumor cell inoculation. One mouse was euthanized when one of its tumors exceeded 12 mm in diameter. The vertical line at 40 days represents the time point when mice with completely regressed tumors were injected i.p. with S180 cells. A one-way ANOVA analysis revealed a significant difference (p =  0.0005) in tumor size between the two groups of mice with either efficient or inefficient AT. Each point represents the mean and SEM. (C) The numbers and the composition of the transferred SR/CR and C57BL/6 (B6) derived leukocytes. There were no significant differences in the numbers of total leukocytes (splenocytes + peritoneal leukocytes) transferred from the SR/CR and C57BL/6 mice (p =  0.969). The numbers of peritoneal immune cells derived from the SR/CR and C57BL/6 mice were not significantly different (p =  0.721). T =  total leukocytes. Per =  peritoneal leukocytes.

**Figure 4:**
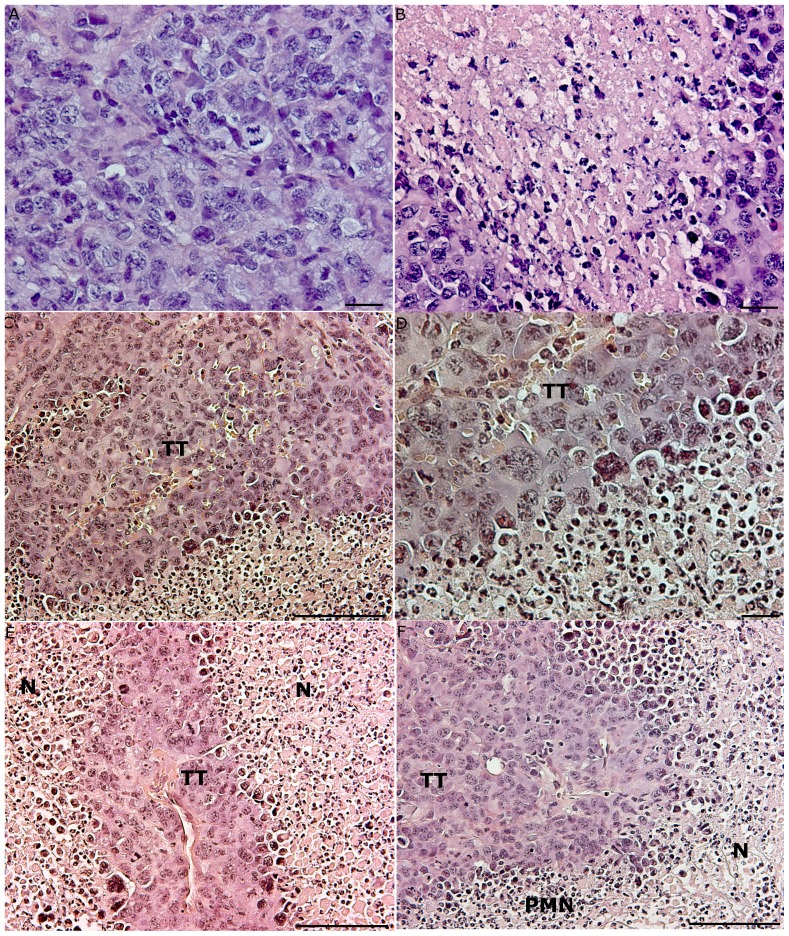
Histology of tumor tissues from mice with or without AT. (A) Fourteen days after inoculation, the tumor tissue was dense with mitotic figures, and no infiltration of immune cells was observed (HE stain, bar 20 µm). (B) A section of tumor tissue (TT) that reached the plateau phase of growth 22 days after inoculation. A large area of necrosis (N) was visible, but no infiltrating immune cells were present (HE stain, bar 20 µm). (C) Massive infiltration of PMNs in S180 tumors after AT of cancer-resistant SR/CR leukocytes to cancer susceptible C57BL/6 mice (representative sections, n = 8). Tumor tissue (TT) from a mouse with tumor regrowth showed large areas of infiltrating PMNs (PMN) and necrosis (N) (HE stain, bar 100 µm). (D) Tumor tissue (TT) from a mouse with tumor regrowth revealed the infiltration of PMNs (PMN) (HE stain, bar 20 µm). (E) The regression of tumor tissue (TT) in a cancer-susceptible C57BL/6 mouse after AT of SR/CR leukocytes. Large necrotic areas (N) in the tumor tissue were observed (HE stain, bar 100 µm). (F) An image of the same tumor as in C), showing a clear demarcation between the vital tumor tissue (TT) and the infiltrating PMNs (PMN), as well as large necrotic (N) areas (HE stain, bar 100 µm).

None of the SR/CR mice developed tumors after the s.c. injection of S180 cells in each axilla (Figure 2). In contrast, all of the susceptible C57BL/6 control mice (n = 12) developed progressively growing tumors that were measurable six days after the s.c. injection of S180 cells (Figure 2), and were euthanized within 30 days. Tumor growth was exponential, and most of the tumors exceeded the maximally allowed size 12–19 days after tumor cell inoculation (Figure 3B). Sections of the tumor tissues exhibited numerous mitotic patterns (Figure 4A). The tumors displayed central areas with necrosis, and no leukocyte infiltrations were observed (Figure 4B).

### AT of SR/CR Leukocytes Significantly Reduced Tumor Size in Susceptible C57BL/6 Mice

Approximately ten days after AT of SR/CR leukocytes to susceptible C57BL/6 mice (n =  8), the tumors began to regress, and 25 days after AT, all of the tumors had regressed substantially (Figure 3B). No residual tumor mass was detectable by inspection or palpation within an observation period of 200 days after the regression. In two mice, however, one of the s.c. tumors began to regrow, and three weeks later, these mice had to be euthanized, after which their tumors were histologically evaluated. In both mice, the tumor tissue contained large areas with necrosis and dense PMN infiltration (Figure 4C–D). None of the remaining six mice that received an i.p. injection of 2×10^6^ S180 cells developed ascites, and these mice remained tumor-free throughout the 250 day observation period (Figure 3B).

The number of leukocytes transferred to each mouse varied by one order of magnitude (Figure 3C). However, there was no correlation between the number of leukocytes transferred and the incidence of tumor re-growth. Complete tumor regression and resistance to the subsequent challenge with S180 cells were observed in several mice that had received smaller numbers of leukocytes than the two mice with tumor re-growth. Peritoneal cells accounted for 12–52% of the transferred leukocytes, and the percentages of transferred peritoneal cells were 30% and 33% in the two mice with tumor re-growth (Figure 3C). Within 20–40 days after AT of the same number of leukocytes from susceptible controls (n =  7), the tumors grew to sizes that required euthanasia in four mice; however, in three mice, the tumors regressed completely (Figure 3A–B). These three mice also resisted a subsequent i.p. injection of S180 cells and remained tumor-free throughout the 250 day observation period. The histological feature observed in all sections of tumors that grew was dense tumor tissue with only small areas of necrosis and no infiltration of immune cells. Susceptible mice receiving AT of leukocytes from SR/CR displayed substantially and statistically significant smaller tumor volumes (Figure 3B, p =  0.0005). In addition, theses mice had marginally and statistically insignificant survival percentages compared to control mice (p =  0.1076).

### AT of SR/CR Leukocytes Depleted of CD4+/CD8+ T Cells and B Cells Significantly Reduced Tumor Sizes in Susceptible Recipients

**Figure 5:**
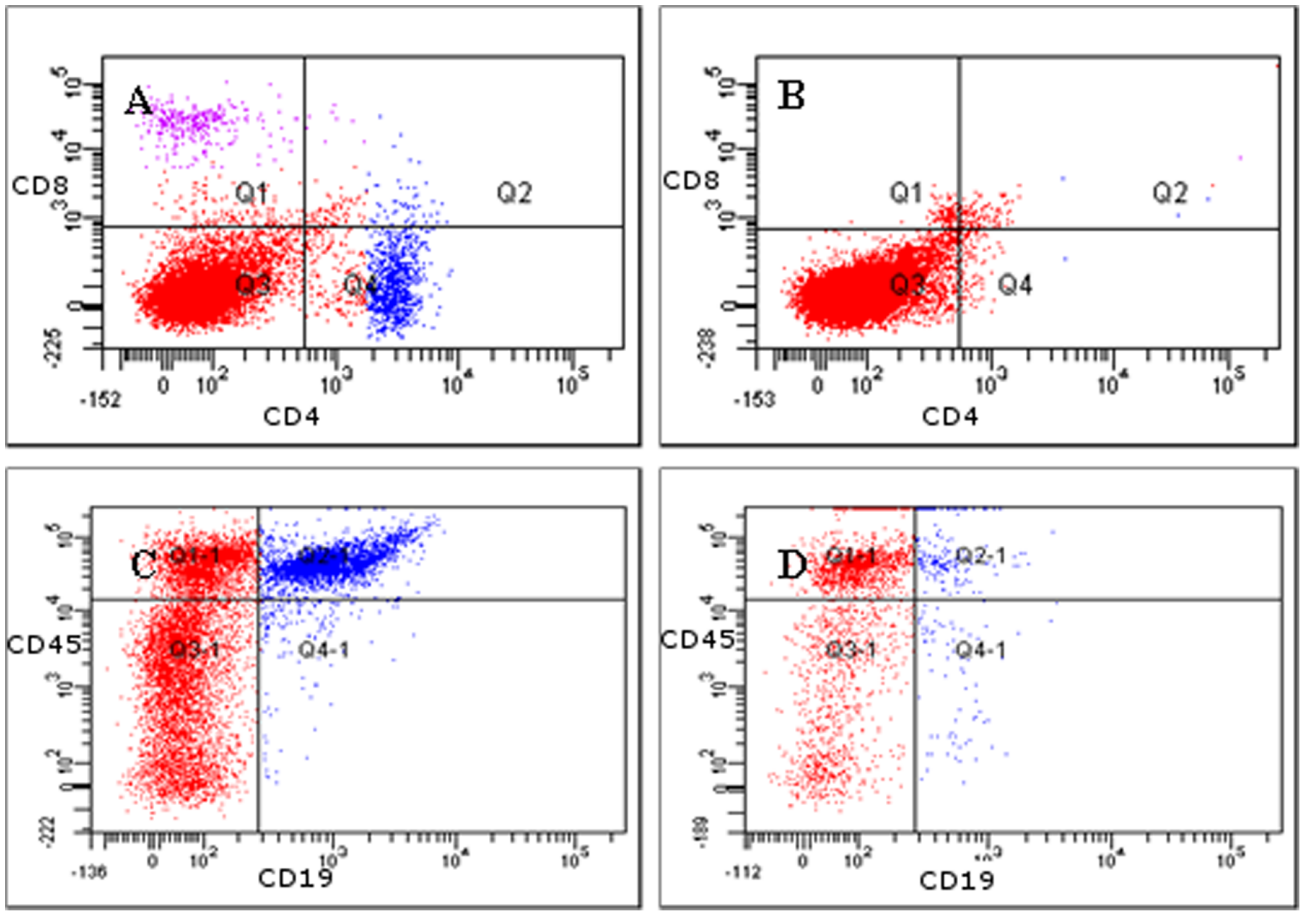
Depletion of T- and B cells, demonstrated by FACS (representative plots, n = 6 in each group). (A) Total leukocytes from a SR/CR mouse prior to depletion, stained for CD8+ (purple) and CD4+ (blue) cells. (B) After depletion, leukocytes were stained with the same antibodies as in A. No CD4+ or CD8+ cells were detected. (C) Total leukocytes from a SR/CR mouse prior to depletion, stained for CD19+ (y-axis) and CD45+ (x-axis) cells. (D) After depletion, leukocytes were stained with the same antibodies as in C. Two percent of the cells were double positive for CD45 and CD19.

**Figure 6:**
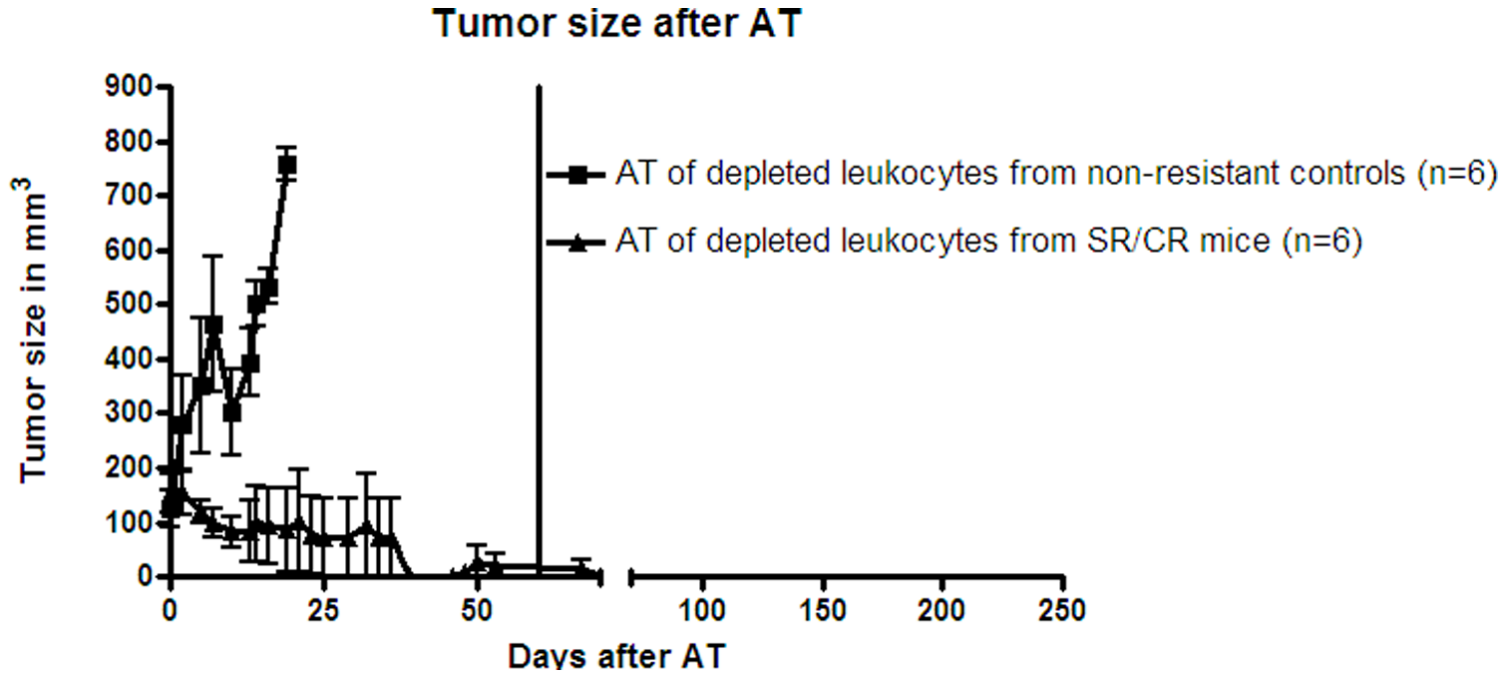
A significant decrease in the S180 tumor volume was observed after AT of SR/CR leukocytes compared to AT of leukocytes from nonresistant controls. Leucocytes were depleted of CD4+/CD8+ T cells and B cells (each point represents the mean and SEM, n =  6 in each group). The tumors in the group that received an AT from nonresistant controls (n =  6) grew exponentially, whereas the tumors in the group that received an AT from SR/CR mice (n =  6) regressed 30–40 days after AT. The tumors in the nonresistant control group were significantly larger (p =  0.0001). The vertical line at 60 days represents the time point when the mice with regressed tumors were injected i.p with S180 cells.

**Figure 7:**
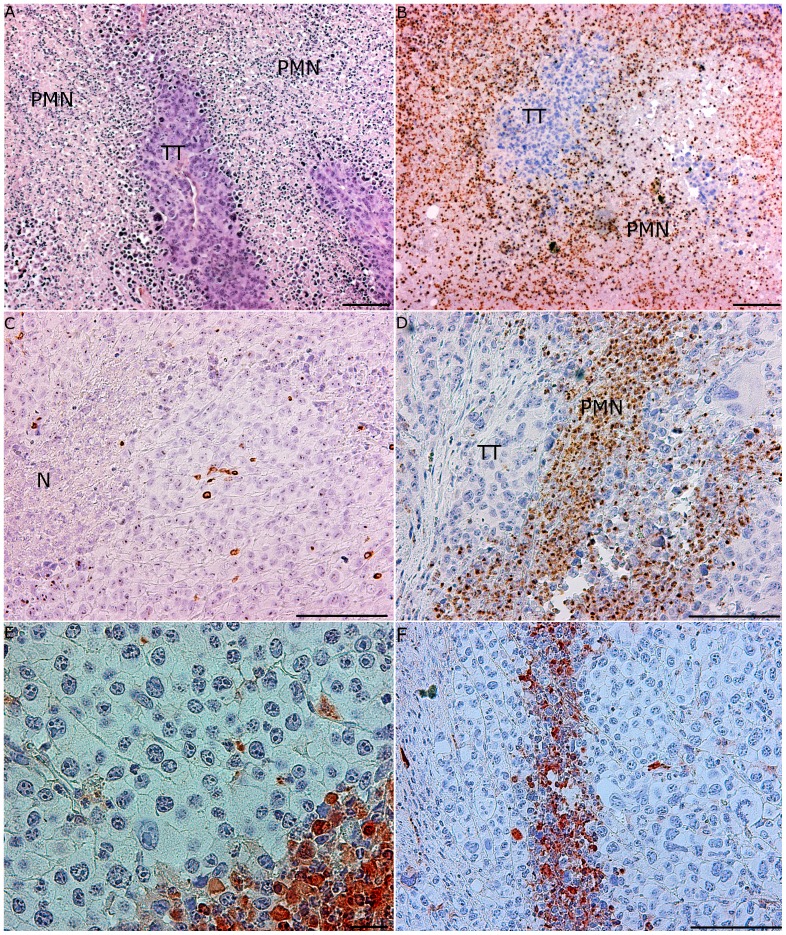
Massive infiltrations of PMNs and apoptotic areas in regressing tumor tissue 35 days after AT of SR/CR leukocytes depleted of CD4+/CD8+ T cells and B cells (representative sections, n =  6). (A) Large necrotic areas with PMN infiltration (PMN) and clearly marked borders between vital and necrotic tumor tissues (TT) (HE stain, bar 100 µm). (B) The necrotic areas showed dense infiltration by PMNs (PMN) (myeloperoxidase staining, bar 100 µm). (C) CD3+ T cells were more sparsely distributed in the tumor tissue and were not found in areas with necrosis (N) (CD3 staining, 20x magnification). (D) PMNs (PMN) infiltrating the tumor tissue (TT) in trabecular formations (myeloperoxidase staining, bar 100 µm). (E) Many of the tumor cells were highly positive for caspase-3 (arrows) indicating apoptosis (caspase-3 staining, bar 20 µm). (F) The distribution pattern of the apoptotic cells had a trabecular formation, which resembled the distribution pattern of the PMNs in the tumor tissue in D (caspase-3 staining, bar 100 µm).

**Figure 8:**
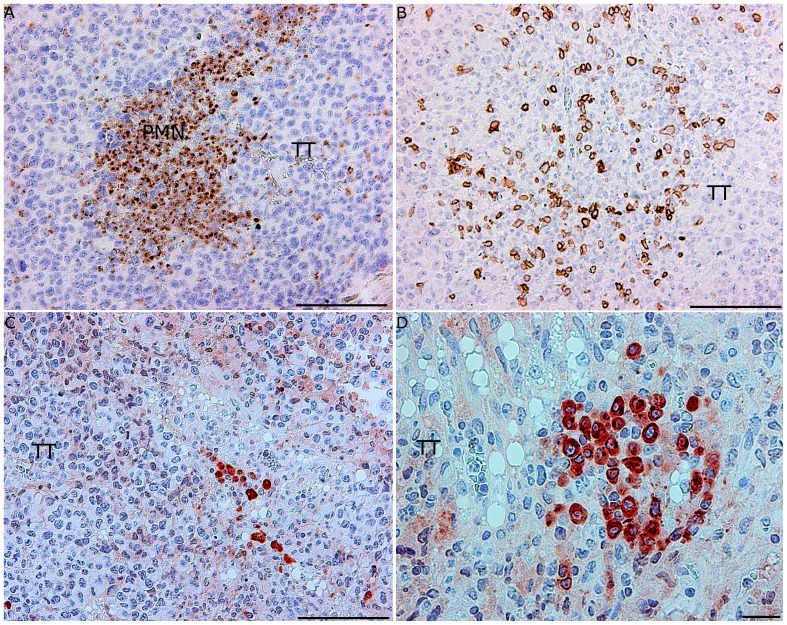
Few infiltrating PMNs or apoptotic areas were observed in growing tumor tissue after AT of leukocytes from nonresistant controls depleted of CD4+/CD8+ T cells and B cells (representative sections, n =  6). (A) Areas exhibiting the infiltration of PMNs (PMN) were observed within the vital tumor tissue (myeloperoxidase staining, bar 100 µm). (B) T cells were densely distributed in the vital tumor tissue (TT) (CD3+ staining, bar 100 µm). (C) Few apoptotic cells were present in the tumor tissue (TT) (caspase-3 staining, bar 100 µm). (D) Apoptotic tumor cells were distributed throughout the vital tumor tissue (TT) (caspase-3 staining, bar 20 µm).

Donor leukocytes were 95–98% depleted of CD4+/CD8+ T cells and B cells (Figure 5). In susceptible mice receiving AT of SR/CR innate immune cells (n =  6) tumors began to regress 8–12 days after the AT, and complete regression was achieved 20–30 days after the AT (Figure 6). These mice were also resistant to the subsequent i.p. injection of 2×10^6^ S180 cells and remained tumor-free throughout the 250 day observation period. In contrast, in susceptible mice receiving AT of purified leucocytes from susceptible controls (n =  6), the tumor size increased continuously, and all mice had to be euthanized 10–20 days after AT due to large tumor burdens (Figure 6). Histology of tumors that regressed after the AT of SR/CR immune cells revealed large areas with apoptotic tumor cells and PMN infiltration (Figure 7A–B). The infiltrating PMNs in the apoptotic tumor tissue displayed a pattern of distribution resembling trabeculae (Figure 7D), which indicated the presence of clear demarcation zones between the vital tumor tissue and the areas with apoptotic tumor cells (Figure 7E–F). Few CD3+ cells were present (Figure 7C), and they were not localized in relation to the apoptotic tumor cells (Figure 7E–F). In tumor tissues from mice receiving AT from susceptible controls, fewer apoptotic areas and fewer PMNs were observed (Figure 8A–D). CD3+ cells were interspersed between the vital tumor cells (Figure 8B).

There was no significant difference between the numbers of innate immune cells transferred from SR/CR mice and susceptible controls (p =  0.7499) (data not shown). The mice that received AT of SR/CR innate immune cells demonstrated significantly higher survival (p<0.0001) and smaller tumor volumes (p< 0.0001) compared to mice that received AT of innate immune cells from susceptible controls (Figure 6).

## Discussion

Within the last decade, the ability of certain cancer cells to evade the immune system has been recognized as a hallmark of these cancer types [9]. Cells from the innate immune system of SR/CR cancer resistant mice have the capacity to circumvent immune evasion under certain experimental conditions [9], [10]. In this study, the cancer resistance mechanism of SR/CR mice appeared to be dependent on interactions between S180 sarcoma cancer cells and SR/CR leukocytes. On the other hand, S180 cells have been shown to have some immune-potentiating properties: the inoculation of irradiated S180 cells along with lethal cancer cells has induced tolerance to the cancer cells in both BALB/c and C57BL/6 mice [11]. Nevertheless, based on the findings reported here, we conclude that SR/CR mice possess a direct and potent anticancer mechanism against S180 cells. The resistance is likely based on the ability of innate immune cells to induce growth arrest and probably apoptosis in S180 solid tumors, as evidenced by the regression of tumors after the AT of innate immune cells from cancer resistant SR/CR donor mice. However, we found no evidence of this potent innate immune cell-based cancer resistance against the EL-4 lymphoma and the J774A.1 monocyte-macrophage cancer cell lines.

Histological sections of the growing tumor tissues displayed central necrotic areas lacking PMNs, and the tumor cells presumably died due to lack of vascularization. Because tumors regressed following AT of leukocytes from cancer resistant mice injected i.p., the transferred leukocytes were likely recruited to the tumor site suggesting an early interplay between the tumor tissue and the donor effector cells from the peritoneal cavity.

When purified innate immune cells were transferred from nonresistant controls, the recipient mice were not rendered resistant to the S180 cancer cell challenge resulting in uncontrolled tumor growth. However, when purified innate immune cells from resistant SR/CR mice were transferred to susceptible recipient mice, the recipients resisted S180 cancer cell growth, resulting in the regression of established tumors (Figure 6) as well as resistance to subsequent i.p. injection of S180 cancer cells. These data suggest that the donor innate immune effector cells had a certain immunological specificity against the S180 cells. However, the regression of some of the tumors in the mice that received AT of total leukocytes from nonresistant controls may have been caused by a reaction of adaptive immune cells, such as specific T-cells, against the S180 cells, as the donor leukocytes had been pre-activated by the injection of irradiated S180 cells 48 hours prior to donation. This putative specific activation of donor T cells cells may have continued in the recipient mice, leading to tumor regression in some individuals (Figure 3A). However, a substantial contribution from innate immune effector cells is more likely as anti-tumor effects were observed after adoptively transferring innate immune cells.

Alloreactivity against the S180 cells cannot explain the regression of the S180 tumors after the AT of SR/CR leukocytes because the tumors did not regress after the AT of leukocytes from nonresistant controls. Previous studies using SR/CR, BALB/c and C57BL/6 mice also support this conclusion SR/CR leukocytes promoted resistance to S180 cells inoculated i.p. or s.c., whereas leukocytes from BALB/c and C57BL/6 mice did not [2], [5], [6]. Innate immune cells from SR/CR mice are presumably functionally different from those from nonresistant controls. There were no significant differences in the numbers of immune cells transferred from SR/CR mice and nonresistant controls after depletion (data not shown).

In tumor tissues from mice that received AT of innate immune cells from the nonresistant controls, some areas of PMN infiltration and apoptosis were observed, but the extent of the infiltration was less pronounced than in the regressing tumor tissue from the mice that received AT of innate immune cells from SR/CR cancer resistant mice. In the latter tissue sections, PMNs were abundantly present in trabecular-like formations in the vital tumor tissue adjacent to apoptotic tumor cells. The presence of CD3+ cells was less pronounced and these cells were not localized to areas of apoptotic tumor tissue in the recipients of anti-cancer innate immune cells from SR/CR mice. In mice that received AT of innate immune cells from nonresistant controls, the presence of CD3+ cells was more extensive, with a diffusive distribution within the tumor tissue. These CD3+ cells were presumably the recipients’ own T cells infiltrating the tumor tissue, and apparently, they did not induce regression of the tumors. The distribution patterns of CD3+ cells and PMNs in tumor tissues differed markedly in relation to the clinical outcome. CD3+ cells were diffusely distributed throughout the tumor tissue, whereas PMNs were localized to necrotic or apoptotic areas. Many tumor cells died by apoptosis, as indicated by the presence of caspase-3 in apoptotic cells [12]. Indeed, the total elimination of tumor cells that occurred following AT of SR/CR innate immune cells in the present work strongly suggest that a large proportion of the cancer cells died via apoptosis caused by the infiltrating PMNs. In this context, SR/CR macrophages have been observed to induce apoptosis in S180 cells [13]. In our experiments, however, PMNs were much more abundant than macrophages in the regressing tumor tissues after AT of SR/CR leukocytes. These PMNs were localized in the border between vital tumor tissue and areas of dead tumor tissue and caspase-3 staining confirmed that the majority of the tumor cells were dying via apoptosis.

In earlier studies, SR/CR mice were resistant to i.p. inoculation with EL-4 and J774A.1 cancer cells. In this study SR/CR mice exhibited no resistance to the EL-4 and J774A.1. When the EL-4 cells were growing as s.c.tumors in SR/CR mice, no PMNs were detected, in contrast to the massive infiltration of PMNs observed in the S180 tumor tissue after AT of SR/CR leukocytes. This finding might be due to cell culture conditions or to the pattern of cancer cell inoculation. On the other hand, such experimental results cast a certain doubt to the robustness of the SR/CR mouse model as a general cancer resistance model. Taken together, we conclude that innate immune cells from SR/CR cancer resistant mice seem to be functionally different from those from nonresistant controls and [14], and the restricted cancer resistance mechanism of SR/CR mice in the present experimental context appeared to be dependent on interactions between S180 cells and SR/CR leukocytes. The data substantiate that growth and spread of cancer cells are dependent on a complex interplay between the cancer cells and the host organism. Here, hereditary components of the innate immune system played a crucial role in this interplay and lead to resistance to an experimental cancer cell line. The fact that leucocytes depleted of adaptive immune cells from the cancer resistant donor mice could be transferred to susceptible recipient mice resulting in complete tumor elimination support the vision of an efficient and adverse event free immunotherapy in future selected cancer types.
